# An Approach for Economic Analysis of Intermodal Transportation

**DOI:** 10.1155/2014/630320

**Published:** 2014-07-24

**Authors:** Bahri Sahin, Huseyin Yilmaz, Yasin Ust, Ali Fuat Guneri, Bahadir Gulsun, Eda Turan

**Affiliations:** ^1^Department of Naval Architecture and Marine Engineering, Yildiz Technical University, Besiktas, 34349 Istanbul, Turkey; ^2^Department of Industrial Engineering, Yildiz Technical University, Besiktas, 34349 Istanbul, Turkey

## Abstract

A different intermodal transportation model based on cost analysis considering technical, economical, and operational parameters is presented. The model consists of such intermodal modes as sea-road, sea-railway, road-railway, and multimode of sea-road-railway. A case study of cargo transportation has been carried out by using the suggested model. Then, the single road transportation mode has been compared to intermodal modes in terms of transportation costs. This comparison takes into account the external costs of intermodal transportation. The research reveals that, in the short distance transportation, single transportation modes always tend to be advantageous. As the transportation distance gets longer, intermodal transportation advantages begin to be effective on the costs. In addition, the proposed method in this study leads to determining the fleet size and capacity for transportation and the appropriate transportation mode.

## 1. Introduction

Intermodal transportation comprises two or more different transportation modes linked end-to-end in order to move cargoes or passengers from the point of origin to the point of destination. Intermodal transport is the term used to describe the movement of goods in one and the same loading unit or vehicle which uses successive, various modes of transport such as road, rail, and seaway. This transportation system has become an important sector in decreasing transport costs and time. The problems in intermodal transport are complicated and involve many parameters to get settings. Thus, intermodal transportation researches have recently increased. Bontekoning et al. [1] made a detailed literature survey on this subject. They reviewed many publications in order to identify the characteristics of the intermodal research community and scientific knowledge base. Another review study was made by Macharis and Bontekoning [2]. They investigated how and which operational research techniques have been applied to support the specific decisions that have to be made by the different decision makers in the intermodal transport system. Tsamboulas et al. [3] presented the development of a methodology with the necessary tools to assess the potential of a specific policy measure to produce a modal shift in favor of intermodal transport. Additionally, their work provides insight into the needs of the decision makers interested in using intermodal transport and examines whether their decisions will have a positive impact on the modal shift towards intermodal transport. Janic [4] developed a model for calculating the full costs of a given intermodal and road transport networks. He concluded that, for the intermodal transport network, the full and internal costs decrease more rapidly with increasing distance in the intermodal case than in the road transport network. Nozick and Morlok [5] described a model developed for medium-term operations planning in an intermodal rail-truck system and developed a very efficient heuristic method. Lozano and Storchi [6] considered an approach using label correcting techniques to find the shortest viable path from an origin to a destination, in a multimodal transportation network. They showed the resulting paths of an application on a network, for different number of modal transfers, and they specified that the choice of a path depends on the user's preferences with respect to costs and the number of modal transfers. Southworth and Peterson [7] described the development and application of a single, integrated digital representation of a multimodal and transcontinental transportation network. Some researchers examined the role of intermodal cooperation and as such differ from the literature on intermodal competition. For example, Givoni and Banister [8] examined the possibility by making the case for aircraft and high speed train substitution under conditions of intermodal integration. They emphasized that society gains from the social and economic benefits of better integrated transport services at lower environmental costs. They concluded that some railway infrastructure should also be seen as a part of the air transport infrastructure. Bookbinder and Fox [9] obtained the optimal routings for intermodal containerized transport from Canada to Mexico. Regan and Golob [10] examined the perceived efficiency of maritime intermodal transfer facilities in California, from the point of view of the trucking companies that use these facilities.

Janic [11] developed an analytical model for the evaluation of performance of long intermodal freight trains and determined the operational, economical, and environmental characteristics of long and conventional intermodal freight trains operating in railway-road intermodal modes of freight transportation system. Additionally, the developed model was applied to intermodal and road transportations systems.

Patterson et al. [12] studied estimation of the potential for reducing the CO_2_ emissions of freight transportation on intermodal services in Canada by applying survey (http://tureng.com/search/questionnaire) to the shippers. They used survey data to develop mode share models. Nine different scenarios for two geographical markets were simulated in order to compare CO_2_ emission ratios of road and intermodal transportation systems.

Bubbico et al. [13] focused on assessing the risk of road and rail transportation cases of hazardous materials transport in Sicily. For each transportation mode, changing route and/or transport modalities and all the combinations of road, rail, and intermodal (road-rail) transport have been calculated and that minimizing the risk has been identified with the aid of the transportation risk analysis tool.

Liao et al. [14] compared road and sea-road intermodal transportation systems in Taiwan using activity based emission modeling. They observed that replacing road transportation with intermodal transportation can significantly reduce the CO_2_ emissions due to the efficiency of marine fuels.

Frémont and Franc [15] investigated combined sea-road transport versus road transport in the region of Le Havre port and Paris. It is shown that intermodal transportation is more suitable in the reference region and also in long distances.

Rizzoli et al. [16] simulated the flow of intermodal terminal units among and within inland intermodal terminals in the scope of platform project. They formed various scenarios in order to evaluate the effects of different technologies and management policies to enhance terminal performances.

Chang [17] studied best routes selection in international intermodal networks. A mathematical model is developed considering multiple objectives, scheduled transportation modes, and demanded delivery times and transportation economies of scale. The model is applied to container freight transportation by road, air, and seaway modes between Taiwan and USA. Additionally, the problem is broken into smaller subproblems with Lagrange relaxation method and solved with Lagrange relaxation technique.

Transport cost is also a significant factor for intermodal transportation for competitiveness of shippers. Calculation and minimization of total transportation costs are required to determine an effective, efficient, and economical transportation system. Unit cargo cost per route length is generally accepted as an indicator of economics.

Many studies have been dedicated in the literature regarding transportation costs of freight and passenger transportation. Ravn and Mazzenga [18] evaluated the quantitative effects of adapting of transportation costs to a trade model and Winebrake et al. [19] and Snaddon [20] studied transportation parameters such as cost, quality, response, flexibility, and dependability comparing of public and private companies. On the other hand, Ozbay et al. [21] studied transportation costs for passengers.

McCann [22], Arnold et al. [23], Dullaerta et al. [24], and Kutanoglu and Lohiya [25] deal with optimality criterion such as, respectively, optimal size of a vehicle or vessel, optimally locating the rail/road terminals for transports, determination of the optimal mixture of transport alternatives to minimize total logistics costs, and presentation of an optimization based model.

There are also several studies in the literature regarding external costs of transportation. For example, Jakob et al. [26] revealed the external (unpaid) and internal (user paid) costs of transport and determined that the external costs were approximately 2.23% of the GDP of Auckland in New Zealand. Panis et al. [27] quantified how large the uncertainty on estimates of road transport externalities is for the future and which parameters are most important for the different modes (passenger cars, heavy duty trucks, buses, and motorcycles).

C. Pilot and S. Pilot [28], Prakash et al. [29], and Al-Khayyal and Hwang [30] focused on minimizing total transportation costs. Al-Khayyal and Hwang [30] also developed a model for finding a minimum cost routing in a network for a heterogeneous fleet of ships.

Macharis et al. [31] presented the effect of fuel prices increase on the market of intermodal transportation terminals with different price scenarios by using a geographical data system for Belgium. They also investigated internalizing the externalities and showed break-even distances for both road and intermodal transportation systems subject to the alteration of fuel prices.

Verma and Verter [32] focused on developing a biobjective optimization model minimizing total transportation cost and total public risk for rail-road intermodal transportation of hazardous materials. In the study, they defined 7 intermodal routes and presented the optimum routes for costs.

Berechman [33] investigated the increase of population in New York and total costs associated with additional traffic, in particular, congestion, safety, and emission. He focused on the estimation of the full marginal costs of truck traffic resulting from the further expansion of the port's activities. In the study, possible solutions such as barge movements, rail expansions, efficient road pricing, demand management, and regulations regarding replacing of old trucks are suggested against truck traffic.

Beuthe et al. [34] presented direct and cross-elasticity estimates of the demands for rail, road, and inland waterways modes of transportation of 10 different groups of commodities and developed a model minimising the generalised cost of transportation tasks.

Transportation modes should be evaluated for several aspects in order to define the total costs extensively. In this context, technical, economical, and operational parameters and externalities such as the costs of the accidents, emissions, and noise should be set clearly for each type of mode considering the probable price escalations during the lifetime of a certain transportation system. For this purpose, in this study, a more realistic cost analysis method, called “the levelised cost analysis method,” is used. Levelised costs are the “ratio of total lifetime expenses versus total expected outputs, expressed in terms of the present value equivalent” according to Nuclear Energy Agency and International Energy Agency [35].

By using this method the economic analysis for passenger transportation seaways has been carried out by Alkan et al. [36]. Recently, the method has been applied to Turkish transportation systems by Sahin et al. [37, 38] for the economic evaluation and comparison of alternative transportation modes. Some studies have also been undertaken with this method for different industries and technologies in the literature. Allan et al. [39] calculated the private levelised costs of two marine energy technologies as wave and tidal stream power for UK electricity generation. Khalaf and Redha [40] have carried out a case study in order to evaluate the levelised unit cost of a new power and water plant. Agashichev [41] also analyzed an integrated cogenerative scheme including gas turbine, unfired heat recovery steam generator, auxiliary boiler, and multistage flash and reverse osmosis comparing the alternatives with the present values of expenses over the economic life of capital and levelised cost of water.

In addition to the above studies in the literature, a different intermodal transportation model based on cost analysis including various technical, economical, and operational parameters is presented in this study. The intermodal modes involved are sea-road, sea-railway, and road-railway as well as multimode of sea-road-railway combinations. A case study of cargo transportation in Turkey has been carried out by using the suggested model. In this study, the analysis is carried out in order to determine the most economic transportation mode in the country for specified routes and a different modelling approach is submitted for intermodal transportation. The selection of vehicle types has been considered with the conditions of the country. Then, the single modal transportation (road, railway, and seaway) has been compared to intermodal modes in terms of transportation costs. This comparison has taken into account the external costs of intermodal transportation. The regional advantages of the transportation modes involved are determined by using the suggested economic model. Besides, the method proposed in this study is thought to contribute to determining the fleet size and the transport capacity and the appropriate transportation mode.

## 2. Modelling of Intermodal Transportation Costs

The cost of transportation is an important selection criterion for determining the appropriate transportation mode. The specific cost defined by the unit freight cost per route length is accepted as an indicator of the transportation cost. In order to calculate the unit transportation cost, all of the factors that influence the cost of transportation have to be taken into consideration. Even though these factors can vary for different transportation modes, basically the main cost components can be classified as capital, fuel, and operational and maintenance costs and external costs such as the cost of accident, emission, and noise. All costs along the lifetime of a vehicle are calculated in certain intervals. Additionally, it is predicted that the amount of cargo carried annually will vary from one year to another. Therefore, cargo transportation cost can be determined utilizing the levelised cost analysis method.

In this section, firstly the intermodal transportation cost model developed by Sahin et al. [37] is presented and its components are given.

### 2.1. Intermodal Transportation Cost Model per Unit of Cargo

Let *U*_*L*_ be total specific cost for each transportation mode such as road, railway, and seaway. For intermodal transportation, the transport cost per unit cargo, (*U*_*K*_), is formulated as shown below by Alkan et al. [36]:

(1) $$\begin{array}{c} U_{K}=X\cdot {\left(U_{L}\right)}_{S}\cdot L_{T}+Y\cdot {\left(U_{L}\right)}_{R}\cdot L_{T}+Z\cdot {\left(U_{L}\right)}_{K}\cdot L_{T}, \end{array}$$

where subscripts *S*,  *R*, and *K* denote seaway, railway, and road, respectively.

The specific intermodal transportation cost per unit cargo and unit route length, (*U*_*KL*_), is

(2) $$\begin{array}{c} U_{KL}=\frac{U_{K}}{L_{T}}=X\cdot {\left(U_{L}\right)}_{S}+Y\cdot {\left(U_{L}\right)}_{R}+Z\cdot {\left(U_{L}\right)}_{K}, \end{array}$$

where *L*_*T*_ is total route length and *X*,  *Y*, and *Z*  (*Z* = 1 − *X* − *Y*) are sharing ratios of seaway, railway, and road in the total route length, respectively. Thus the intermodal transportation cost can be realized as follows.

 If *X* = 0, then railway-road intermodal mode is realized.
 If *Y* = 0, then seaway-road mode is realized.
 If *Z* = 0, then seaway-railway mode is realized.
 If *X* > 0,   *Y* > 0, and *Z* > 0, then road-railway-seaway mode is realized.

In addition, handling and stocking costs should be considered according to the type of the intermodal transportation. Levelised handling and stocking costs per unit cargo can be described as follows.

*Handling Cost per Unit Cargo *(*U*_YB_)

(3) $$\begin{array}{c} U_{\text{YB}}=\frac{\zeta C_{\text{yb}}\sum_{t=1}^{n}{\left(\left(1+e_{\text{yb}}\right)/\left(1+r\right)\right)}^{t}}{\left(1+e_{\text{yb}}\right)\sum_{t=1}^{n}{\left(1+r\right)}^{-t}}. \end{array}$$

*Stocking Cost per Unit Cargo *(*U*_*D*_)

(4) $$\begin{array}{c} U_{D}=\frac{\psi \;C_{d}\sum_{t=1}^{n}{\left(\left(1+e_{d}\right)/\left(1+r\right)\right)}^{t}}{\left(1+e_{d}\right)\sum_{t=1}^{n}{\left(1+r\right)}^{-t}}, \end{array}$$

where *C*_yb_ is the net present value of handling price per unit cargo, *e*_yb_ is annual escalation rate in handling price, *ζ* is additional handling number required for intermodal transportation, *C*_*d*_ is net present value of the daily stocking price per unit cargo, *e*_*d*_ is annual escalation rate in stocking price, Ψ is the number of stocking days, *n* is average economic lifetime, and *r* is discount rate.

Using a generalized and an easily applicable method for all transportation modes, levelised investment, *U*_*c*_, operation and maintenance, *U*_*m*_, fuel, *U*_*f*_, and external costs, *U*_ex_, per cargo related to the technoeconomic and operational parameters are described below, respectively. 

*Investment Cost per Unit of Cargo *(*U*_*c*_). Investment, operational and maintenance, fuel and lubricant, and external costs per unit of cargo have been calculated with the economical model presented by Sahin et al. [37, 38].

The unit cargo investment cost, *U*_*c*_, can be found as shown below:

(5) Uc={∑t=1nIc[(1−((t−1)/n))i+(1/n)](1+r)−t}[2L+VsZsa]2YkYd  Vs(8760−Zbt−  Zbk)∑t=1n(1+r)−t.

In the above formula *I*_*C*_ represents investment cost including infrastructure, *L* is route length, *V*_*s*_ is service speed of vehicle, *Z*_sa_ represents waiting time between sequential trips, *Y*_*k*_ is cargo capacity of vehicle, *Y*_*d*_ shows fullness ratio of vehicle, *Z*_bt_ is annual maintenance-repair time, *Z*_bk_ is annual idle time, and *i* represents interest rate. 

*Operational and Maintenance Costs per Unit of Cargo *(*U*_*m*_). Consider

(6) Um={∑t=1n[Cmo(1+em)t+(sIc(1−(t/n)))(1+es)t](1+r)−t}×[2L+VsZsa]2YkYdVs(8760−Zbt−Zbk)∑t=1n(1+r)−t

where *C*_mo_ is annual operation and maintenance costs, *e*_*m*_ is escalation rate for future operational and maintenance costs, *s* is insurance percentage (%*I*_*c*_),  and *e*_*s*_ is escalation rate for future insurance cost. 

*Fuel and Lubricant Costs per Unit of Cargo *(*U*_*f*_). Fuel and lubricant costs per unit of cargo, *U*_*f*_, can be shown as

(7) $$\begin{array}{c} U_{f}=\frac{\left(B_{f}P_{f}+B_{o}P_{o}\right)L\sum_{t=1}^{n}\left[{\left(1+e_{f}\right)}^{t}{\left(1+r\right)}^{-t}\right]}{\left(Y_{k}Y_{d}\right)\left[\sum_{t=1}^{n}\left[{\left(1+r\right)}^{-t}\right]\right]}, \end{array}$$

where *B*_*f*_ is fuel consumption per km (main + aux.), *P*_*f*_ is fuel price, *B*_*o*_ is lubricant consumption per km (main + aux.), *P*_*o*_ is lubricant price, and *e*_*f*_ is escalation rate for future fuel cost.

*External Costs per Unit of Cargo.* The external costs per unit of cargo can be formulated as

(8) $$\begin{array}{c} U_{\text{ex}}=\frac{\left(c_{\text{ac}}+c_{p}+c_{n}\right)\;L\sum_{t=1}^{n}{\left(\left(1+e_{x}\right)/\left(1+r\right)\right)}^{t}}{\left(1+e_{x}\right)\sum_{t=1}^{n}\left[{\left(1+r\right)}^{-t}\right]}\left(\frac{Y_{d}^{*}}{Y_{d}}\right). \end{array}$$

In the above formula, *c*_ac_,   *c*_*p*_, and *c*_*n*_ are specific cost of accidents, the specific cost of pollution caused by emission, and the specific cost of pollution caused by noise, respectively. *Y*_d_* is reference fullness ratio used for the calculation of specific external costs, while *e*_*x*_ is the escalation rate in the external costs.

According to above formulas, total transportation cost per cargo, *U*_*K*_, is shown below:

(9) $$\begin{array}{c} U_{K}=U_{c}+U_{m}+U_{f}+U_{\text{ex}}\;\left(\text{\$}/\text{ton}\right)\; \end{array}$$

and then specific cost, *U*_*KL*_, becomes

(10) $$\begin{array}{c} U_{KL}=\frac{U_{K}}{L_{T}}\;\left(\text{\$}/\text{ton}\cdot \text{km}\right).\; \end{array}$$

*Equivalent Infrastructure Investment Cost per Vehicle in Road Transportation.* In road transportation, the sharing of infrastructure investment costs per vehicle subject to vehicle types can be defined as below:

(11) $$\begin{array}{c} {\left(ICT\right)}_{j}=\frac{IC_{L}\sum_{i=1}^{K}L_{i}}{\sum_{j=1}^{M}N_{j}g_{j}}\;g_{j}. \end{array}$$

Equivalent infrastructure investment cost for a unit length of road, *IC*_*L*_, can be represented with the below equation

(12) $$\begin{array}{c} IC_{L}=\frac{\sum_{i=1}^{K}I_{i}L_{i}}{\sum_{i=1}^{K}L_{i}}, \end{array}$$

where *i* represents road type (highway, state way, and province way), *K* represents the number of road types, *I*_*i*_ is infrastructure investment cost per unit length of related road type, *L*_*i*_ is the length of related road/railway type, *j* is vehicle type, *M* is the number of vehicle types, *N*_*j*_ is the number of vehicles in related vehicle category, and *g*_*j*_ is the sharing factor of infrastructure investment cost for the related vehicle types [38]. Sharing of annual cost of road maintenance and repair subject to vehicle types is shown as below:

(13) $$\begin{array}{c} {\left(C_{B}\right)}_{j}=\frac{C_{\text{BT}}\lambda_{j}}{\sum_{j=1}^{M}N_{j}\lambda_{j}}, \end{array}$$

where *C*_BT_ is annual maintenance and repair cost and *λ*_*j*_ is the wear and tear factors for the road surface for the related vehicle type or the sharing percentage of the costs which are attributed to maintenance and repair by Sahin et al. [38]. The number of vehicles per vehicle type, the types of vehicles, and equivalent factor in road transportation are given in Table 1.

In this section, the calculations are valid for only road transportation; however, similar applications can be derived for railway transportation mode.

## 3. A Case Study: An Application of Cost Analyses for Intermodal Transportation in Turkey

A cost analysis is performed and costs are compared by using data of Turkey for different modes of transportation including intermodal transportation in this study. Therefore, current data for each transportation mode are considered for the proposed cost analysis method. The interest rate, *i*, is assumed to be 8%, the discount rate, *r*, is 10%, the escalation rate for future fuel costs, *e*_*f*_, is 5%, the escalation rate for future operational and maintenance costs, *e*_*m*_, is 3%, and the escalation rate for future external costs, *e*_*x*_, is 3% for all transportation modes. Data and studies on external cost estimations for different transportation modes on country basis are not satisfactory. Therefore, taking into consideration the available data for Turkey by Sahin et al. [38] and the results of different international analyses such as those carried out by Forkenbrock [42, 43], Beuthe et al. [44], and Quinet [45, 46], estimations are made for the specific external cost data shown in Table 2 for different transportation modes. In this study, standard vehicle types that can be used in this country are selected for different transportation modes. These vehicles are a general cargo ship with a net cargo capacity of 2970 tons for the sea transportation, a cargo train with a capacity of 700 tons for the railway and a truck with a capacity of 20 tons for the road. The technical and economical parameters of the vehicles are summarized in Table 3. The parameters in Table 3 have been taken from the Ministry of Transport, Maritime Affairs and Communications of Republic of Turkey, Turkish State Railways, and several maritime and highway logistics companies. The data have not been based on a statistical study. They have been collected by interviews.

The outcomes of cost analysis using the economic model proposed in this study and considering different transportation modes are presented below.

### 3.1. Cost Analyses for Sea-Road Intermodal Transportation

**Figure 1:**
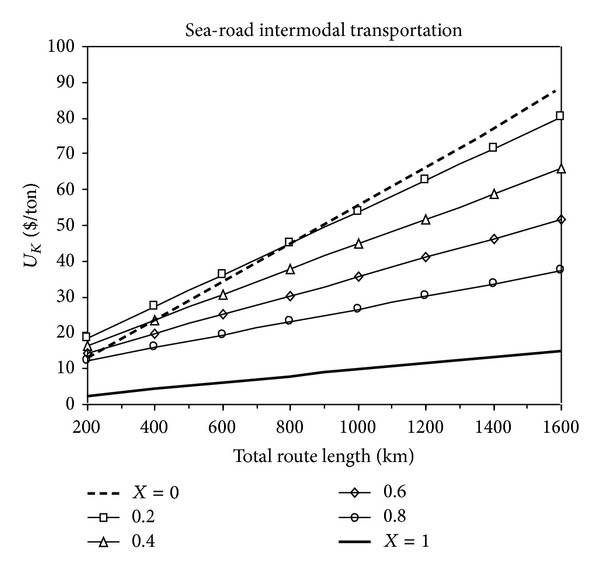
The change of total transportation cost with the total route distance for sea-road intermodal transportation.

The change in the total transportation costs with the total route distance for sea-road intermodal transportation is shown in Figure 1. In these figures *X* = 0 condition refers to the single road transport, *X* = 1 refers to single sea transport, and 0 < *X* < 1 condition refers to the sea-road intermodal transportation.

As the proportion of sea transportation distance to the total transportation distance increases, the total cost of transportation decreases and becomes minimum when *X* = 1, which indicates single sea transportation. If the single sea transportation is unavailable, sea-road intermodal transportation is considered. The comparison of the total cost of sea-road intermodal transportation with single road transportation is seen in Figure 1. Under *X* = 0 line this figure also shows the economic feasibility area of sea-road transportation over that of road transportation. Thus, it can be seen from Figure 1 that the economical superiority of single road transportation according to sea-road intermodal transportation is only valid when the distance does not exceed 200 kilometres.

### 3.2. Cost Analyses for Sea-Railway Intermodal Transportation

**Figure 2:**
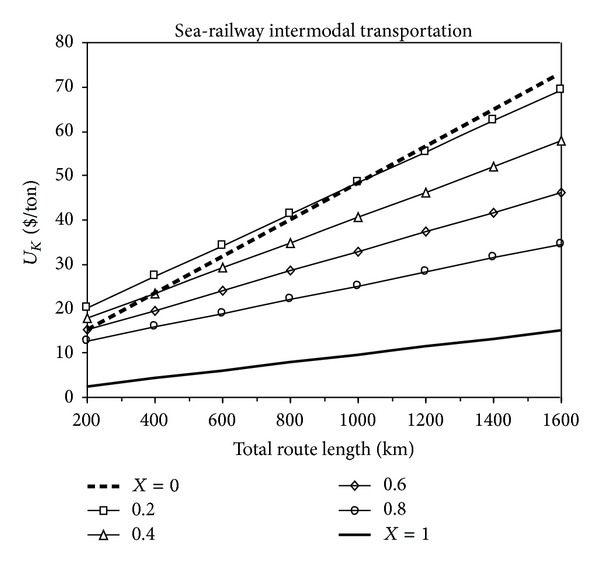
The change of total transportation cost with the total route distance for sea-railway intermodal transportation.

The results of economic analysis for the intermodal transportation modes are presented in Figure 2. In these figures, the costs of sea-railway transportation (0 < *X* < 1) and single sea transportation (*X* = 1) and single railway transportation (*X* = 0) are compared. It can be seen from Figure 2 that when the single sea transportation is not available in the routes, it is more appropriate to use the sea-railway intermodal transportation under the line *X* = 0. Furthermore, this figure indicates that if the total route distance is over 1000 km, then the sea-railway intermodal transportation is always more economic than the single railway transportation.

### 3.3. Cost Analyses for Railway-Road Intermodal Transportation

**Figure 3:**
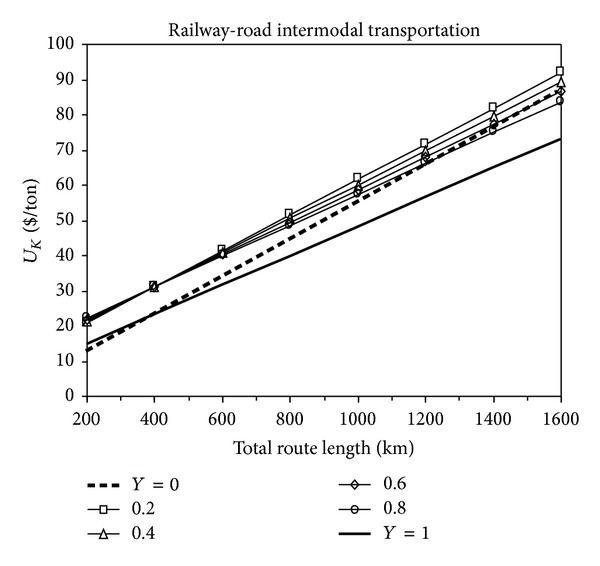
The change of total transportation cost with the total route distance for railway-road intermodal transportation.

The change in total transportation costs with the total route distance for railway-road intermodal transportation is shown in Figure 3. In this figure, *Y* = 0 condition refers to the single road transport, *Y* = 1 condition refers to the single railway transportation, and 0 < *Y* < 1 condition refers to the railway-road intermodal transportation. It can be clearly seen from Figure 3 that the single railway transportation is more economic than railway-road intermodal transportation for all given routes. However, it can also be seen from the same figure that graph (*Y* = 0) exceeds graph (0 < *Y* < 1) after the intersection of these two graphs at *L* = 1200 km. This comparison between single road transportation (*Y* = 0) and railway-road intermodal transportation (0 < *Y* < 1) from Figure 3 points out that the railway-road intermodal transportation is more economic than the single road transportation for route distances exceeding 1200 km. That means the railway-road intermodal transportation is economically more feasible than the single road transportation when the route distance is greater than 1200 km. Furthermore, the same figure states that if the total route distance is more than 380 km, the single railway transportation (*Y* = 1) is more economic than the single road transportation (*Y* = 0). It is clear that additional handling costs are determinant factors in the low-cost intermodal transportation.

### 3.4. Cost Analyses for Sea-Railway-Road Multimodal Transportation

**Figure 4:**
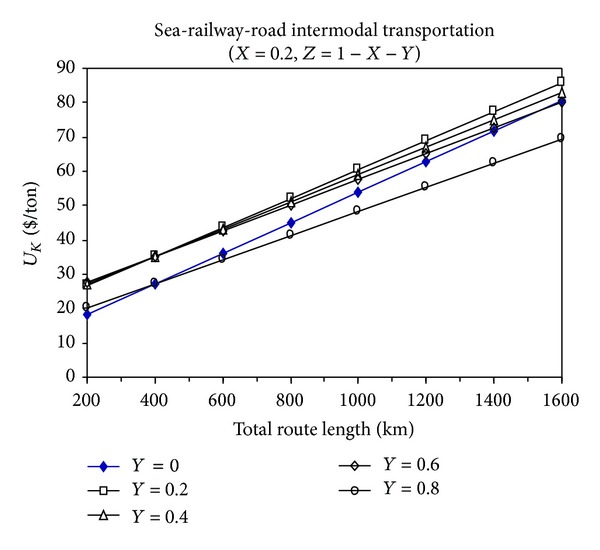
The change of total transportation cost with the total route distance for sea-railway-road multimodal transportation (*X* = 0.2).

**Figure 5:**
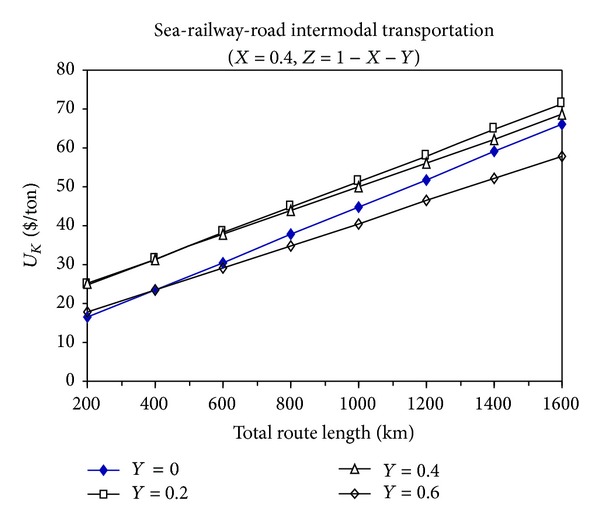
The change of total transportation cost with the total route distance for sea-railway-road multimodal transportation (*X* = 0.4).

**Figure 6:**
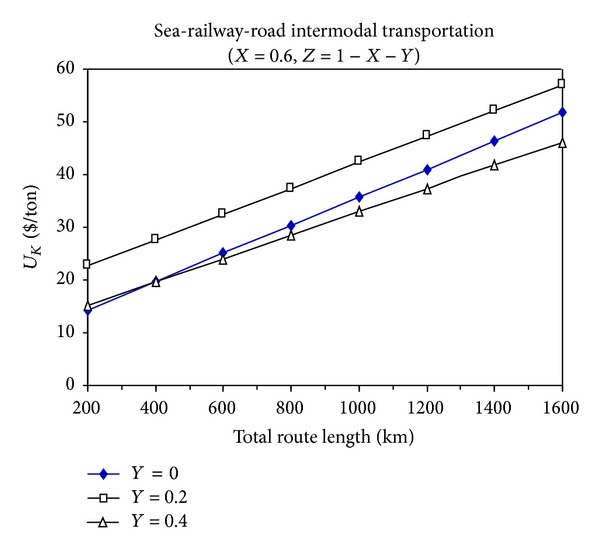
The change of total transportation cost with the total route distance for sea-railway-road multimodal transportation (*X* = 0.6).

Figures 4, 5, and 6 show the total cost changes in sea-railway-road multimodal transportation in proportion to their variable use in a specific total route distance.

From these figures, it can be seen that the multimodal transportation is not economic compared to the intermodal transportation modes, and it is not appropriate to use multimodal transportation unless it is absolutely avoidable.

When the sea-road intermodal transportation is compared to the sea-railway intermodal transportation in terms of economical feasibility, it can be seen that their superiority over each other is determined by the proportion of sea transportation distance (*X*) to the total transportation distance. For example, when *X* is equal to 0.2, then *L* is greater than 400 km and then the sea-railway intermodal transportation is more economic than the sea-road intermodal transportation.

## 4. Discussions and Conclusion

In this study, a different intermodal transportation model based on cost analysis including various technical, economical, and operational parameters is presented. These intermodal modes are sea-road, sea-railway, and road-railway as well as multimode of sea-road-railway. In addition, a case study of cargo transportation in Turkey has been carried out by using the suggested model with basic formulas covering all cost items. The selection of vehicle types takes into consideration the conditions of the country.

Evaluating the results of the economical analysis of different intermodal transportation systems, the cases listed below have been ascertained.

- When the total route distance exceeds 200 km, the sea-road intermodal transportation becomes more economic than the single road transportation, and it starts to get full economical after 800 km.
- When the total route distance is more than 1000 km, then the sea-railway intermodal transportation is always more economic than the single railway transportation.
- The railway-road intermodal transportation becomes more economic than single road transportation when the route distance is greater than 1200 km. When the total route distance is less than 380 km, the single road transportation is more economic than the single railway transportation.
- Intermodal transportation systems of sea-road and sea-railway are more economic in comparison with railway-road transportation for all route distances. This result indicates that railway-road transportation should be preferred in the lines when there is no opportunity of using sea transportation.
- In comparison with intermodal transportation, sea-railway-road multimode transportation is not economical and should not be preferred if not necessary.

The mostly utilized mode in cargo transportation in Turkey is road transportation and the ratios of road transportation movements are more excessive than other countries which are in the same economical level. It is definitely shown in the study that railway and seaway transportation modes are more economical compared to road transportation in some distance lengths. The intermodal transportation considering the combinations of different modes is also advantageous subject to cost effectiveness, sustainable environment, and so on. The study is significant since it clearly points out that adoption of alternative transportation systems instead of single road transportation would be more convenient in Turkey.

We generally concluded that, in the short distance transportation, single transportation modes always tend to be advantageous. As the transportation distance gets longer, intermodal transportation advantages begin to be effective. The single sea transportation mode is always more economical than all other modes, while road transportation is more advantageous than the railway in short distances; especially for the transportation systems possessing sea modes, intermodal transportation in short distances is more advantageous. In the intermodal road-railway transportation, single transportation modes are more advantageous than the intermodal transportation modes. The reason is that additional handling cost increases the intermodal transportation costs to a great extent in short distance transportation. In other words, in the low-cost single and intermodal transportation, additional handling costs are determinant factors.

This study is thought to contribute to determining the optimal fleet size and suitable vehicle capacities for different modes of transportation considering the annual cargo potential and fullness ratio of the routes. The considered model is hoped to improve the transport service quality conditions in the future works of intermodal transportation systems.

## Figures and Tables

**Table 1:** The number of vehicles, equivalent factor, and wear and tear factor for road surface with respect to vehicle type.

Vehicle type	Number of vehicles	Equivalent factor	Wear and tear factor
Automobile (*j* = 1)	*N*_1_ = 4,784,140	*g*_1_ = 0.15	*λ*_1_ = 0.00086
Minibus (*j* = 2)	*N*_2_ = 249,041	*g*_2_ = 0.25	*λ*_2_ = 0.071
Bus (*j* = 3)	*N*_3_ = 126,587	*g*_3_ = 0.50	*λ*_3_ = 0.143
Light truck (*j* = 4)	*N*_4_ = 1,073,728	*g*_4_ = 0.25	*λ*_4_ = 0.071
Truck (*j* = 5)	*N*_5_ = 412,881	*g*_5_ = 1.00	*λ*_5_ = 0.285
Articulated lorry (*j* = 6)	*N*_6_ = 61,965	*g*_6_ = 2.00	*λ*_6_ = 0.428

**Table 2:** Specific external costs (*c*_ac_, *c*_*p*_, *c*_*n*_).

Environmental effects	Road	Railway	Seaway
	$/(ton*·*km)	$/(ton*·*km)	$/(ton*·*km)
Accident (*c*_ac_)	3.3 × 10^−3^	4 × 10^−4^	6 × 10^−5^
Emission (*c*_*p*_)	4.5 × 10^−4^	1.1 × 10^−4^	3.85 × 10^−4^
Noise (*c*_*n*_)	2.2 × 10^−4^	1.5 × 10^−4^	—

**Table 3:** Technical and economical properties of the vehicles.

Description of vehicles	Symbol	Unit	Transportation modes		
			Seaway	Railway	Road
			General cargo ship	Freight train	Truck
Investment cost including infrastructure	*I*_*c*_	$	6 000 000	6 500 000	90 000
Average economic lifetime	*n*	Year	20	20	10
Insurance percentage (%*I*_*c*_)	*s*	$	0.020	0.00923	0.02778
Service speed of vehicle	*V*_*s*_	km/h	22	35	50
Cargo capacity	*Y*_*k*_	Ton	2970	700	20
Annual maintenance-repair time	*Z*_bt_	Hours	300	1200	720
Annual idle time	*Z*_bk_	Hours	1095	1095	5110
Fuel consumption per km (main + aux.)	*B*_*f*_	Litre/km	16	7	0.3
Lubricant consumption per km (main + aux.)	*B*_*o*_	Litre/km	0.11	0.05	0.0036
Fuel price	*P*_*f*_	$/litre	0.3	1.70	1.70
Lubricant price	*P*_*o*_	$/litre	1.10	5.50	5.50
Annual operation and maintenance costs	*C*_mo_	$/year	750 000	800 000	15 000
Interest rate	*i*		0.08	0.08	0.08
Discount rate	*r*		0.1	0.1	0.1
Escalation rate for future operational and maintenance costs	*e*_*m*_		0.03	0.03	0.03
Escalation rate for future fuel cost	*e*_*f*_		0.05	0.05	0.05
Escalation rate for future insurance cost	*e*_*s*_		0.03	0.03	0.03
Escalation rate for future external costs	*e*_*x*_		0.03	0.03	0.03
Route length	*L*	km	1000	1000	1000
Waiting time between sequential trips	*Z*_sa_	Hours	9.00	30	6.00
Specific cost of accident	*c*_ac_	$/(ton*·*km)	6.00*E* − 05	4.00*E* − 04	3.30*E* − 03
Specific cost of pollution	*c*_*p*_	$/(ton*·*km)	3.85*E* − 04	1.10*E* − 04	4.50*E* − 04
Specific cost of noise	*c*_*n*_	$/(ton*·*km)	0.00*E* + 00	1.50*E* − 04	2.20*E* − 04
Average fullness ratio	*Y*_*d*_		0.70	0.90	0.90
Reference fullness ratio for the specific external costs	*Y*_d_*		0.70	0.70	0.70

Note: all values in the tables are considered data of March 2006.
